# Embolic brain infarction related to posttraumatic occlusion of vertebral artery resulting from cervical spine injury: a case report

**DOI:** 10.1186/1752-1947-8-344

**Published:** 2014-10-14

**Authors:** Yaoki Nakao, Hiroshi Terai

**Affiliations:** 1 Department of Emergency Medicine, Daiyukai General Hospital, 1-9-9, Sakura, Ichinomiya 491-8551, Japan

**Keywords:** Brain infarction, Cervical spine injury, Traumatic vertebral artery injury

## Abstract

**Introduction:**

The frequency of vertebrobasilar ischemia in patients with cervical spine trauma had been regarded as low in many published papers. However, some case reports have described cervical spine injury associated with blunt vertebral artery injury. Many aspects of the management of vertebral artery injuries still remain controversial, including the screening criteria, the diagnostic modality, and the optimal treatment for various lesions. The case of a patient who had a brain infarction due to recanalization of his occluded vertebral artery following open reduction of cervical spinal dislocation is presented here.

**Case presentation:**

A 41-year-old Asian man presented with C4 to C5 distractive flexion injury manifesting with quadriplegia and anesthesia below his C3 cord level (including phrenic nerve paralysis), and bowel and bladder dysfunction. Magnetic resonance angiography and computed tomography angiography showed left extracranial vertebral artery occlusion and patent contralateral vertebral artery. He was observed without antiplatelet and/or anticoagulation therapy, and underwent surgery (open reduction and internal fusion of C4 to C5, and tracheostomy) 8 hours after the injury. After surgery, supraspinal symptoms such as left horizontal nystagmus and left homonymous hemianopsia led to cranial computed tomography and magnetic resonance imaging, which showed left-side cerebellar infarction in his posterior inferior cerebellar artery territory and right-side posterior cerebral artery infarction. Magnetic resonance angiography and computed tomography angiography demonstrated patent bilateral vertebral artery (but hypoplastic right vertebral artery) and occluded right posterior cerebral artery. His injured vertebral artery was treated conservatively, which did not cause any other ischemic complications.

**Conclusions:**

The management of asymptomatic vertebral artery injury is controversial with several treatment options available, including observation alone, antiplatelet therapy, anticoagulation therapy, or invasive intervention. Although there are some reports in which management with observation alone is described as safe, we should pay serious attention to the vertebral artery injury caused by cervical spine trauma.

## Introduction

Various nonpenetrating injuries, as well as penetrating injuries, have been associated with cerebrovascular injuries, not excepting cervical spine fracture and dislocation [1]. Compared with carotid arterial injuries, vertebral artery (VA) injuries associated with blunt cervical spine trauma were thought to be infrequent because most of them were asymptomatic and because imaging studies were not performed routinely. Recent improvements in imaging technology and increased use of screening protocols have led to a greater number of these injuries being identified [1]. Traumatic VA injury can have disastrous consequences of basilar territory infarction and death [2]. Although it is often assumed that the reduction of a fracture without treatment of an associated asymptomatic VA injury is safe [3], when the vertebrobasilar ischemia occurs, the mortality rate is from 75% to 86% [4]. VA injuries are relatively frequent and may be associated with significant morbidity and mortality in patients with cervical spine fractures or dislocations [2]. Foremost in dealing with such an unusual but potentially devastating injury is determining whether the injury can be detected and effectively treated before complications occur.

Despite such a situation, well-defined treatment recommendations are still lacking [1]. We present the case of a patient who had a brain infarction due to recanalization of his occluded VA following closed reduction and open fixation of cervical spinal dislocation, and discuss the management of asymptomatic VA injuries associated with spine trauma.

## Case presentation

A 41-year-old right-handed Asian man was attacked from behind by a cow and he tumbled down. He received a hyperflexion injury when he was struck on the top of his head. He did not lose consciousness but described an immediate loss of power and sensation in both his arms and legs. He was triple immobilized and transferred to the local hospital where cervical spine trauma was suspected. There were no findings from a cranial computed tomography (CT) scan to suggest brain infarction. The unavailability of spine specialists prompted the patient’s transfer. He arrived at our institution 6 hours after injury.

Clinical assessment in our hospital found a Glasgow Coma Scale of 15 out of 15 but complete neurologic deficits below the level of C5. Plain X-ray film images obtained at this time showed a C4 on C5 dislocation. Further information regarding the severity of his injury was required, and so a cervical CT scan and magnetic resonance imaging (MRI) were performed.

**Figure 1:**
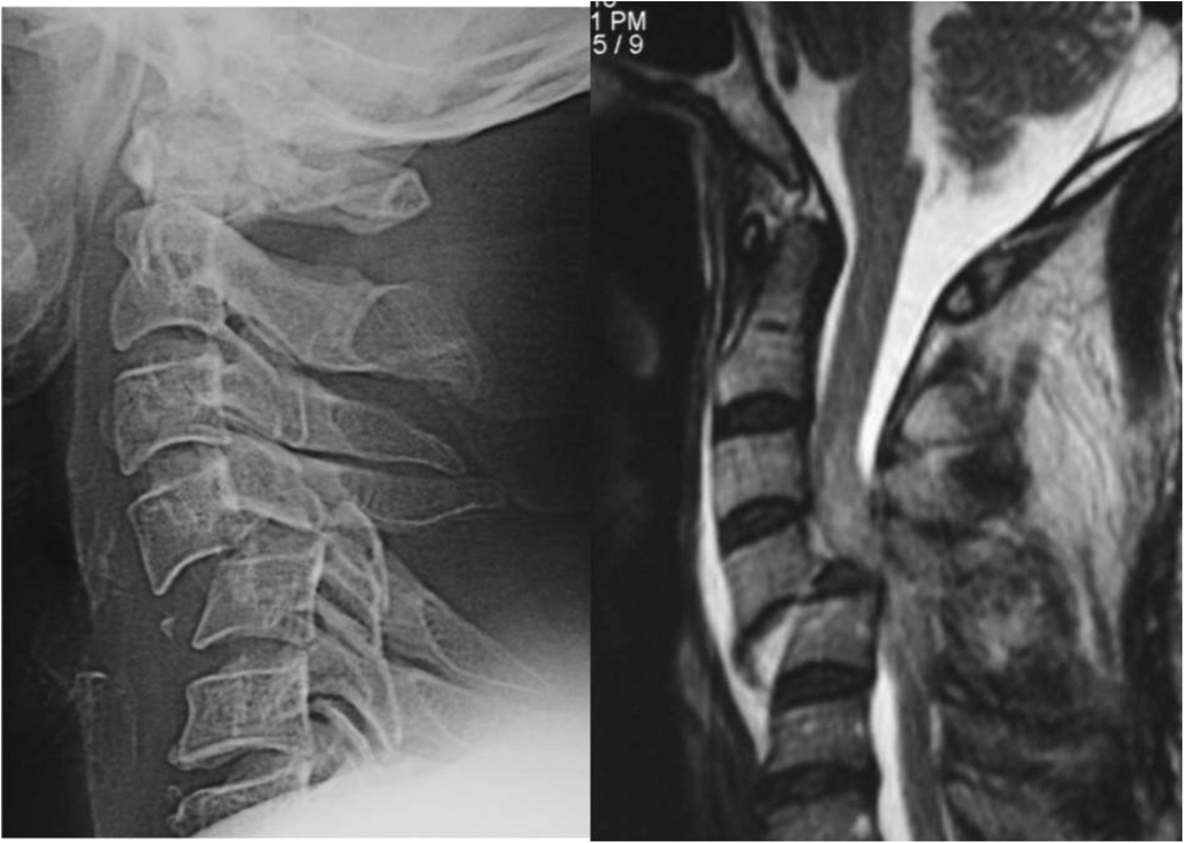
Preoperative imaging study of the cervical spine.

**Figure 2:**
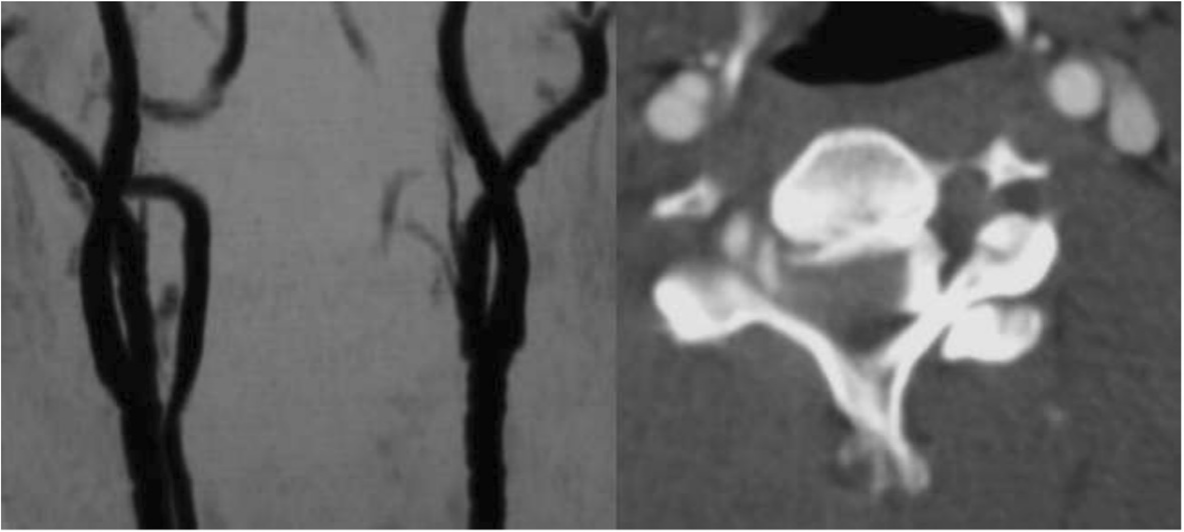
Preoperative angiography showing vertebral artery injury.

A CT scan showed a bifacet dislocation with over 50% displacement of C4 on C5 (Figure 1), and the MRI revealed a severely compressed dural tube at C4 to C5 level and high signal intensity in his spinal cord on T2-weighted imaging (Figure 2). A sagittal CT showed bilateral dislocation of facet joints. Magnetic resonance angiography (MRA) showed complete occlusion of his right VA.

**Figure 3:**
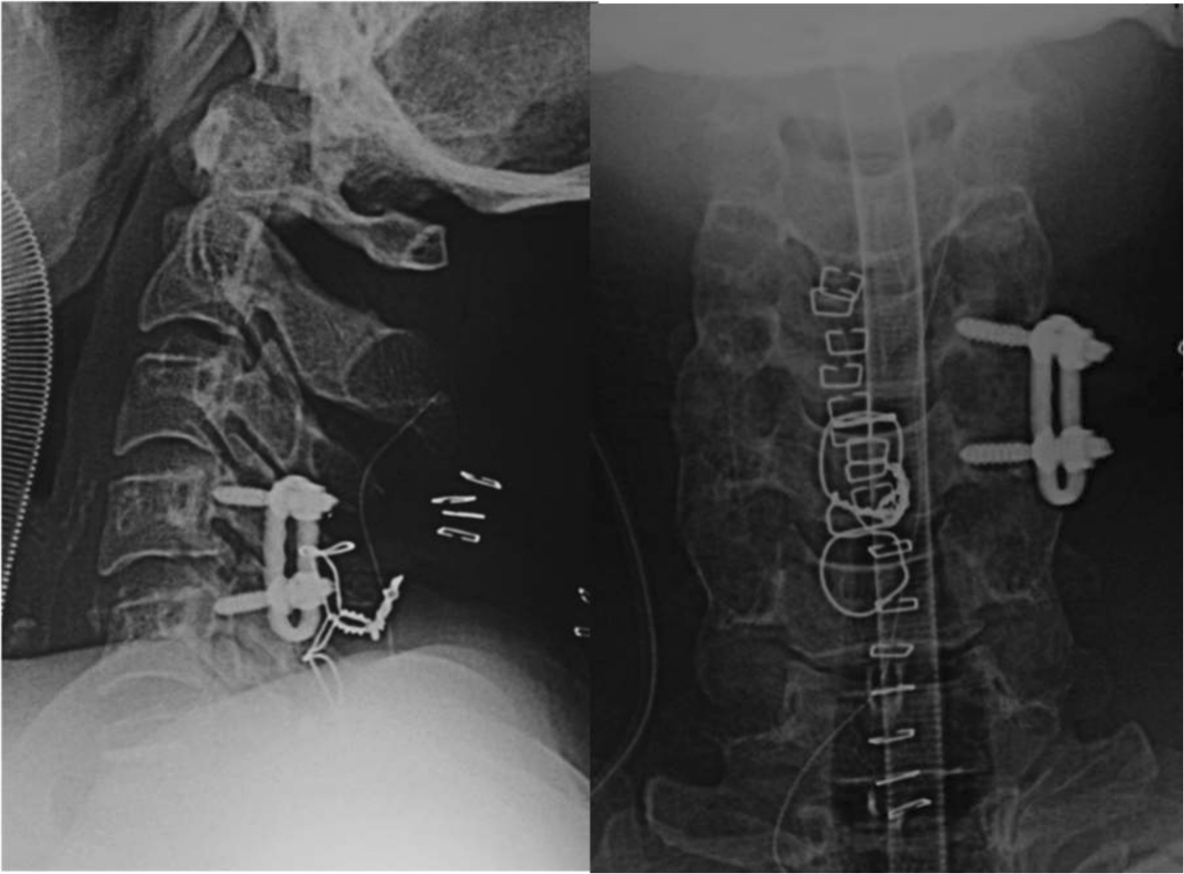
Postoperative radiography of the cervical spine.

He was taken to the operating room 100 minutes after arrival. We performed closed reduction and posterior open surgery under general anesthesia. We used pedicle screws on the left side and spinous process wiring for fixation, and local bone was grafted around his C4 to C5 facet joints bilaterally. Plain X-ray film images obtained just after the surgery showed good fixation (Figure 3).

**Figure 4:**
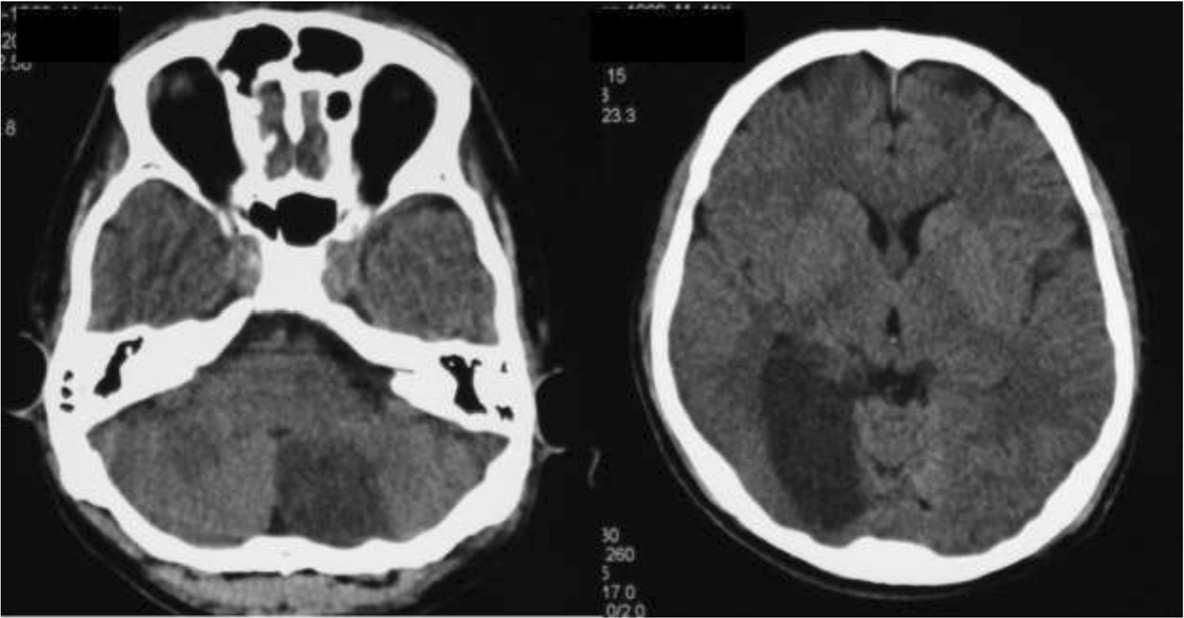
Postoperative brain computed tomography.

**Figure 5:**
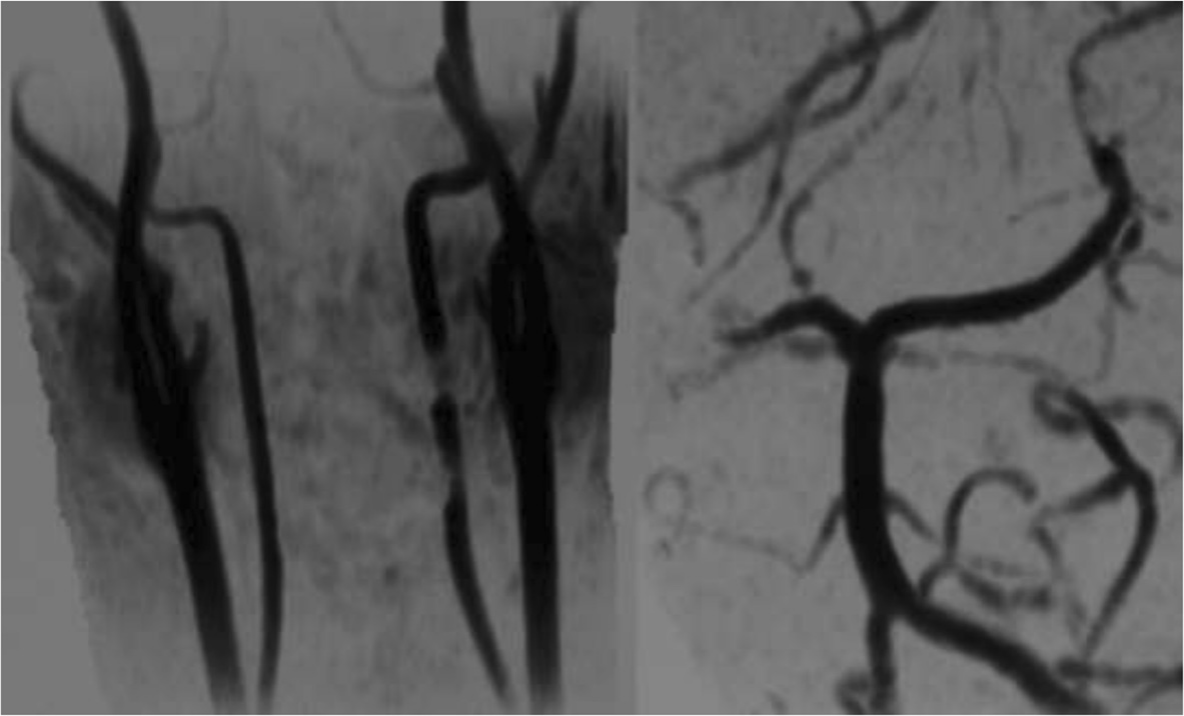
Postoperative magnetic resonance angiography.

He demonstrated consciousness deterioration associated with repeated vomiting and left hemianopsia. These symptoms led us to examine his brain. There were low dense lesions in his right posterior lobe and left cerebellum (Figure 4). MRA showed occlusion of his right posterior cerebral artery and recanalization of his occluded left VA (Figure 5). This seemed to be due to distal embolization of the clot around the occluded vessel. We did not administrate tissue plasminogen activator because actual onset was unknown and because he was on the intensive perioperative management. Left hemianopsia has remained although there was no additional event.

## Discussion

We present the case of a 41-year-old man with a bilateral cervical facet joint dislocation associated with unilateral VA injury, treated by closed reduction and posterior fixation. After surgery, he demonstrated an episode of brain infarction. In this case, brain infarction seemed to be caused by artery-to-artery embolization that originated from recanalized VA. Perhaps, VA occlusion on the dominant side caused by cervical spinal dislocation led to cerebellar infarction in his posterior inferior cerebellar artery territory due to hemodynamic compromise or arterial dissection.

The incidence of VA injury ranges from 0.075% to 0.77% of blunt trauma admissions [1,4,5]. When asymptomatic patients are screened for blunt cerebrovascular injuries, the incidence rate rises to 1% of all patients with blunt trauma and as high as 2.7% in patients with an Injury Severity Score >16 [6,7]. The possibility of association with cerebrovascular injury rises to approximately 17.2 to 25.5% in ‘high risk’ patients who satisfy the Denver screening criteria for blunt cerebrovascular injury [8,9].

Some reports [10,11] have described patients with cervical spinal trauma who had rapidly fatal neurologic disorders suggestive of serious vertebrobasilar insufficiency. In fact, the time course of vertebrobasilar ischemia secondary to VA occlusion is variable, ranging from a few hours to several years. In our case, the time course was 24 hours after the injury and 12 hours after the reductive surgery, respectively.

Vessel occlusion may be acute or delayed, secondary to damage to the intimal lining with delayed platelet aggregation, followed by thrombus formation and occlusion [3,12]. Direct injury from a surrounding structure such as a bony tip also may result in VA injury. An external layer injury may occur and cause a pseudoaneurysm and vessel tear in patients from greater force [13]. In most cases, the V2 segment that runs through the vertebral foramen of C6 until C2 is affected [8,13,14], and the vascular lesion usually presents next to the cervical fractures because of its fixation to the spine within the transverse foramen from C1 through to C6. The most frequent site of the VA lesion is C5 to C6 [3]. The type of fracture associated with VA injury is variable. In particular, two types of cervical fractures are associated with higher risk for VA injury respectively: (a) fractures involving the transverse foramen; (b) subluxations. These types constitute 46% to 75% of VA injuries associated with cervical trauma [15,16].

A high clinical index of suspicion remains the most important factor in making the diagnosis [17]. In patients suspected to have a lesion of the VA after cervical trauma who have altered consciousness, dysarthria, blurred vision, nystagmus, ataxia, or dysphagia, cerebrovascular examination should be indicated [18]. Once a patient is proven to be at risk of VA injury, we should take imaging studies to rule out VA injury and avoid neurological deterioration, even if there are no stigmata of vertebrobasilar insufficiency.

Although there have been no definite standards or guidelines as to what is the most important and optimal imaging study for patients with a suspected VA injury, a broad-scale, less invasive, low-cost, and fast screening program is probably the best way to diagnose lesions [7]. MRA is quite accurate in the detection of near or total occlusion of the V2 segment noninvasively [13,15,18-20]. However , slow blood flow in the small vessels on MRA can be confused with occlusion as a result of insufficient resolution [21]. Conventional angiography is superior to MRA in the detection of nonoccluded intimal disruption, which occasionally causes distal embolization; however, it is not performed as the first-line diagnostic procedure because of its invasiveness [20]. There are some papers regarding computed tomography angiography (CTA) with more than 16-slice scanners as to be equal to conventional angiography in terms of sensitivity [19,22,23]. In addition, CTA is more widely available, has fewer contraindications, and provides greater spatial resolution than MRA. CTA may become an acceptable alternative to conventional angiography. We willingly indicate multidetector CTA in patients with ‘high risk’ cervical spine fractures.

Treatment of blunt VA injury remains controversial. Reported treatments for asymptomatic VA injury include supportive management, surgical ligation, radiologic embolization, systemic heparinization, and antiplatelet therapy [24,25]. Management strategies proposed are based on the radiological grade and clinical severity of cervical spine trauma and VA injury, but are still not standardized and vary from anticoagulation to ligation and thrombectomy.

The most frequently used specific treatment is anticoagulation. There have been several studies attempting to evaluate the impact of antithrombotic agents on the progression or development of sequelae of blunt cerebrovascular injuries. A series of retrospective studies [13,15,26] found that administration of antithrombotic agents reduces the rate of neurologic sequelae after blunt cerebrovascular injuries. The rationale for administration of anticoagulant and/or antiplatelet has been to: 1) minimize clot formation at the site of intimal injury; 2) decrease further propagation of the clot which has formed, allowing the internal fibrinolytic systemic to dissolve the clot; and 3) prevent embolization. However, systemic administration introduces risks of hemorrhagic complications, particularly in multisystem trauma victims. We can reduce the incidence of bleeding complications by avoiding bolus heparin dosing and targeting a lower partial thromboplastin time [4]. Although there has not been a direct controlled comparison of heparinization versus antiplatelet agents in the prevention of cerebral vascular event after VA injury, several studies performed subgroup analysis in an attempt to address this question.

There has been discussion of preemptive embolization of blunt VA injury as primary therapy. This is based on the premise that occlusion of one VA is generally well tolerated. The presence of VA agenesis has been found to be from 1.8% to 3.1% in autopsy study [27] and unilateral VA dominance is uncommon. Hoshino *et al.*[28] performed unilateral VA ligation on 15 patients without adverse sequelae on long-term follow-up. However, routine embolization introduces the risk that the contralateral VA may acquire transient flow disturbances, resulting in infarction. There have also been several preliminary studies that have indicated the safety and feasibility of catheter-directed therapy to include embolization of pseudoaneurysms and stenting of intimal injuries [29].

We gave priority to cervical fixation over treatment of asymptomatic VA injury although, of course, we understood the risk of distal embolization. That is because we tried to decompress the neural structure and stabilize the cervical spine promptly to prevent neurological deterioration and enable early implementation of rehabilitation, and because we intended to avoid bleeding complication in the case that a tracheostoma might be required following the cervical fixation. Perhaps we could achieve reduction and fixation by taking proximal occlusion interventionally. The ideal treatment of patients with cervical spine injuries associated with VA injury might include the help of an interventionist.

## Conclusions

The management of asymptomatic VA injury is controversial with several treatment options available. Although there are some reports in which management with observation alone is described as safe, we should pay serious attention to the VA injury caused by cervical spine trauma. The ideal treatment of patients with cervical spine injuries associated with VA injury whose cervical spine needs immediate fixation might include the help of an interventionist.

## Consent

Informed consent was obtained from the patient for publication of this case report and accompanying images. The document was written by his wife because the patient could not make a signature by himself due to severe tetraplegia. A copy of the written consent is available for review by the Editor-in-Chief of this journal. The protocols for human procedures used in this study were approved by the ethics committee of our institution.

## Competing interests

The authors declare that they have no competing interests.

## Authors’ contributions

Both authors are the main health care providers for this patient. Both authors read and approved the final manuscript.
